# Accuracy of intraoperative frozen section for the evaluation of ovarian neoplasms: an institutional experience

**DOI:** 10.1186/s12957-016-0849-x

**Published:** 2016-03-31

**Authors:** Atif Ali Hashmi, Samreen Naz, Muhammad Muzzammil Edhi, Naveen Faridi, Syed Danish Hussain, Shazia Mumtaz, Mehmood Khan

**Affiliations:** Department of Histopathology, Liaquat National Hospital and Medical College, Karachi, Pakistan; Liaquat National Hospital and Medical College, Karachi, Pakistan; Dhaka Medical College, Dhaka, Bangladesh

**Keywords:** Ovarian cancer, Frozen section, Borderline ovarian tumors

## Abstract

**Background:**

Ovarian neoplasms are a heterogeneous group of tumors including surface epithelial, germ cell and sex cord stromal tumors with a subset having low malignant potential (borderline tumors). While the surgical management plan differs in different categories of tumors, preoperative diagnosis is seldom available. In these circumstances, the role of frozen section becomes invaluable. In the current study, we aimed to evaluate the accuracy of the frozen section of ovarian tumors in our setup.

**Methods:**

It was a retrospective study involving 141 cases of ovarian tumors undergoing surgical resection with frozen section evaluation from January 2009 to December 2014. After gross examination, one to five blocks were prepared on the frozen section depending upon the size of the specimen. After frozen section reporting, specimens were processed routinely for final paraffin section evaluation. Results of frozen and paraffin sections were categorized in benign, borderline, and malignant, and accuracy of frozen section was determined.

**Results:**

Out of 141 cases, 107 were diagnosed as benign on final (paraffin) examination, while 6 were borderline and 28 were malignant. Out of 107 benign cases, 45 were non-neoplastic cystic lesions of the ovary including endometriotic, follicular, and corpus luteal cysts. The most common benign neoplastic tumor was mature cystic teratoma (20 cases) followed by mucinous cystadenoma (19 cases), serous cystadenoma (14 cases), sex cord stromal tumors (8 cases), and Brenner tumor (1 case). Among borderline cases, four cases were serous and two of mucinous neoplasms. The most common malignant neoplasm was serous carcinoma (11 cases) followed by mucinous carcinoma (6 cases). The overall accuracy of frozen section diagnosis is above 99 %. The sensitivity and specificity for benign tumors were found to be 100 and 97 %, respectively. The sensitivity and specificity for borderline tumors was 83 and 99 %, respectively, while for malignant tumors, it was 96 and 100 %, respectively.

**Conclusions:**

We found a high sensitivity and specificity of frozen section for the diagnosis of ovarian tumors and to determine its malignant potential. Therefore, it should always be used when the preoperative diagnosis is not definite to govern extent of surgical resection. However, under-diagnosis can occur in tumors of borderline category which can be minimized by increased sampling on the frozen section.

## Background

In modern day oncologic surgeries, use of the frozen section as an intraoperative guide for surgeons is gaining popularity. The technique was devised a little more than 100 years ago and has advanced in terms of technical expertise and improved microscopic optics. While the role of the frozen section in certain oncologic surgeries like evaluation of margins in squamous cell carcinoma of head and neck and sentinel lymph node evaluation in breast cancer is deemed essential, its role is still a matter of debate in ovarian surgeries [1–3].

Ovarian neoplasms are a heterogeneous group of tumors including surface epithelial tumors, germ cell tumors, sex cord stromal tumors, and secondary malignancies. The management plan for germ cell tumors even if malignant differs from surface epithelial tumors in respect of conservative surgical approach and preservation of fertility. For surface epithelial tumors, the surgical option differs for benign, borderline, and malignant tumors. In malignant cases, staging laprotomy is performed which includes total abdominal hysterectomy with bilateral salpingo-oophorectomy, pelvic, and para-aortic lymph node sampling (up to renal vein level), peritoneal sampling (multiple peritoneal biopsies and peritoneal cytology) and omentectomy. Benign tumors are generally managed with removal of ovarian cyst or mass only (cystectomy) especially for young women who want to preserve fertility. On the other hand, a conservative approach is usually followed in borderline tumors with limited omental sampling and omission of lymph node dissection if they are clinically not enlarged. Fertility sparing options can also be adopted in young patients [4]. Despite of diverse surgical management plans for different categories of tumors, preoperative diagnosis is not possible in all cases, especially those with normal serum markers. In these situations, intraoperative frozen section becomes crucial as a means of guiding surgical management especially in young females where preservation of fertility is desired. The accuracy of frozen section for ovarian malignancies varies from institution to institution and is generally considered to be accurate in more than 70 % of cases. The aim of the current study is to determine the accuracy of the frozen section for ovarian tumors in our setup.

## Methods

**Fig. 1:**
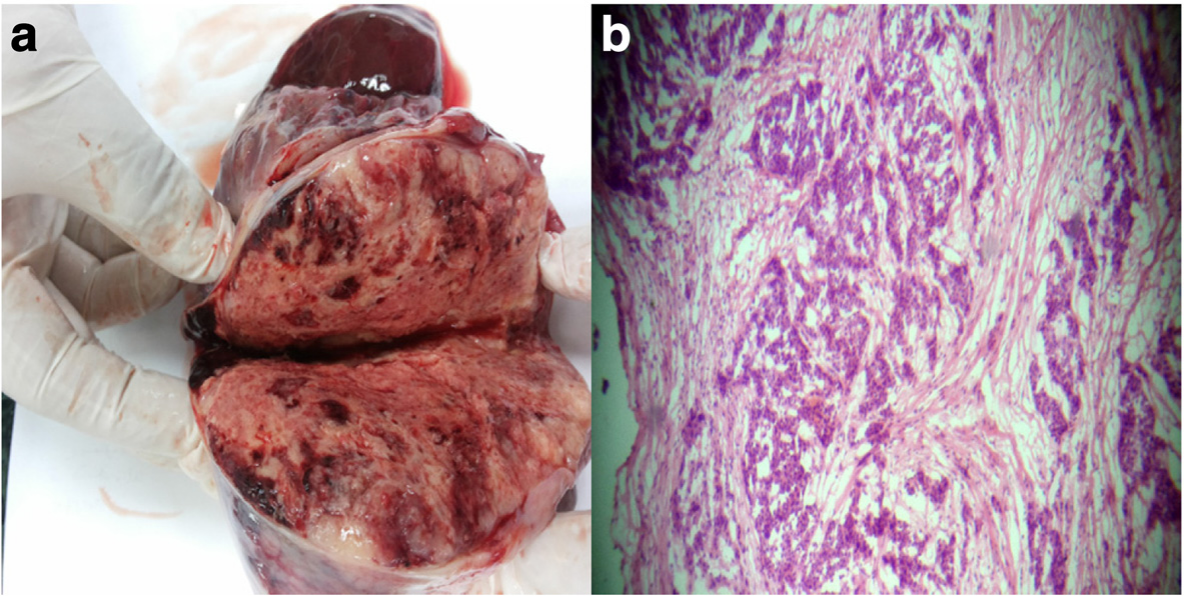
**a** Gross specimen of the ovary for the frozen section. **b** Microscopic section of serous carcinoma of ovary for frozen section

It was a retrospective study involving 141 cases of suspected ovarian masses in which the frozen section was requested from January 2010 to December 2014. The specimens were received in the histopathology department of Liaquat National hospital. After gross examination including tumor size, capsular integrity and presence of solid and cystic areas, frozen section blocks were prepared. The number of blocks ranged from one to five with an average of two. Two to four slides were prepared from each block and stained with hematoxylin and eosin stains (Fig. 1). One touch preparation was also made. After reporting of frozen section, the tissue was routinely processed for paraffin blocks. Frozen sections were reported by one of the three different senior pathologists with more than 5 years experience of reporting gynecologic pathology. Similarly paraffin sections were also reported by one of these three pathologists but not the one who reported the frozen section of that particular case so that one specific case was reported by two different pathologists, one reporting the frozen section and the other reporting the paraffin section, respectively. The reports of frozen and paraffin sections were divided into benign, borderline and malignant. The sensitivity, specificity, positive predictive and negative predictive values were determined separately for benign, borderline, and malignant cases by 2 × 2 tables considering final paraffin section diagnosis as gold standard. For benign tumors, a benign diagnosis on the frozen section is considered true negative and any diagnosis of malignancy even borderline on the frozen section is considered false positive. For borderline cases, borderline diagnosis on the frozen section is considered true positive while benign diagnosis is taken as false negative. Finally for malignant cases, a concordant diagnosis on frozen section is taken as true positive, while a benign or borderline diagnosis is considered false negative.

## Results

A total of 141 cases were sent for frozen section evaluation during the study period, out of which 107 were diagnosed as benign on final (paraffin) examination, whereas 6 were borderline and 28 were malignant. Out of 107 benign cases, 45 were non-neoplastic cystic lesions of the ovary including endometriotic, follicular, and corpus luteal cysts. The most common benign neoplastic tumor was mature cystic teratoma (20 cases) followed by mucinous cystadenoma (19 cases), serous cystadenoma (14 cases), sex cord stromal tumors (8 cases), and Brenner tumor (1 case). Among borderline neoplasms, 4 cases were of borderline serous and 2 cases of borderline mucinous neoplasms. The most common malignant neoplasm was serous carcinoma (11 cases) followed by mucinous carcinoma (6 cases) as shown in Table 1. There were 3 cases which were diagnosed as infiltrating adenocarcinomas, NOS. These were high grade undifferentiated tumors which could not be further categorized into serous endometrioid or any other category after application of extensive panel of immunohistochemistry including WT1, ER, PR, and P53.

**Table 1:** Distribution of ovarian lesions evaluated by frozen section

		Benign frequency	Borderline frequency	Malignant frequency
Benign non-neoplastic cysts	Endometriotic cyst	21	0	0
	Paratubal cyst	4	0	0
	Abscess	5	0	0
	Follicular cyst	3	0	0
	Corpus luteal cyst	8	0	0
	Chronic granulomatous inflammation	4	0	0
Surface epithilial tumors	Serous tumors	14	4	11
	Mucinous tumors	19	2	6
	Endometroid carcinoma	0	0	3
	Clear cell carcinoma	0	0	1
	Brenner tumor	1	0	0
	Transitional cell carcinoma	0	0	1
	Adenocarcinoma, NOS	0	0	3
	Malignant mixed mullerian tumor	0	0	1
Germ cell tumors	Dysgerminoma	0	0	1
	Teratoma	20	0	1
Sex cord stromal tumors	Adult granulosa cell tumor	4	0	0
	Fibroma thecoma	4	0	0

Table 2 shows the comparison of frozen and paraffin section diagnosis. The two cases with incorrect diagnosis include borderline mucinous neoplasm and serous adenocarcinoma which were under-diagnosed as benign mucinous cystadenoma and borderline serous tumor on the frozen section. Table 3 shows that the overall accuracy for benign, borderline, and malignant tumors is above 99 %. The sensitivity and specificity for borderline tumors is 83 and 99 %, respectively, while for malignant tumors is 96 and 100 %, respectively. Cohen’s Kappa was run to determine if there was agreement between two pathologists’ judgment. The results showed that there was strong agreement between the two pathologists’ judgments, *κ* = 0.989, *p* < 0.05.

**Table 2:** Comparison of frozen and paraffin section diagnosis for ovarian neoplasms

		Final (paraffin) diagnosis		
		Benign frequency	Borderline frequency	Malignant frequency
Frozen section diagnosis	Benign	107	1	0
	Borderline	0	5	1
	Malignant	0	0	27

**Table 3:** Sensitivity, specificity, positive predictive value, negative predictive value, and accuracy of the frozen section for ovarian neoplasms

	Benign	Borderline	Malignant
Sensitivity	100.00 %	83.30 % (77.14–89.46 %)	96.40 % (93.33–99.47 %)
Specificity	97.10 % (94.33–99.87 %)	99.30 % (97.92–100 %)	100.00 %
Positive predictive value	99.10 % (97.54–100 %)	83.33 % (77.18–89.48 %)	100.00 %
Negative predictive value	100.00 %	99.30 % (97.92–100 %)	99.12 % (97.58–100 %)
Accuracy	99.30 % (97.92–100 %)	99.58 % (98.51–100 %)	99.30 % (97.92–100 %)

## Discussion

Ovarian cancer is the third most common malignancy in this part of the world, and its incidence is on the rise especially in young age women [5]. A subset of high-grade serous carcinomas is associated with BRCA mutations. Whether this rising incidence of ovarian carcinoma in this region is linked to BRCA mutations is yet to be fully understood as BRCA mutations are thought to be prevalent in women with breast cancers in this region [6]. Frozen section evaluation of ovarian tumors is a very useful approach to individualize surgical intervention and conserve fertility when needed and to avoid overtreatment.

The accuracy of the frozen section for ovarian tumors varies among different institutions. Subbian et al. in a study involving 135 cases of ovarian tumors found an overall accuracy of 84.2 %. They found the lowest sensitivity for borderline tumors (31.2 %) especially of the mucinous category [7]. On the other hand, Supraset et al. in a review of 112 cases revealed a sensitivity of 100, 84, and 92 %, respectively, for benign, borderline, and malignant tumors [8]. In another retrospective review of 282 cases, sensitivities of the frozen section for benign, borderline, and malignant tumors were 97.5, 95.8, and 95.6 %, and corresponding specificities were 97.5, 97.6, and 100 %, respectively. They found the lowest positive predictive value in borderline group (79.3 %), all of them with mucinous type epithelium as described by other authors [9]. Another oncology center reported an overall accuracy of 91.85 %. The relative sensitivities were 99.2, 88.46, and 82.95 %, respectively, for benign, borderline, and malignant tumors. There were 18 false negative diagnoses; most of them belonged to the borderline group [10]. A few authors specifically focused on borderline ovarian tumors. Gultekin et al. evaluated 82 cases of borderline ovarian tumors, 42.7 % of which were of mucinous histology. The concordance with final diagnosis was found in 69.5 % cases. The rates of over-diagnosis and under-diagnosis were 1.2 and 29.3 %, respectively. They proposed that tumor size, presence of solid component, and preoperative CA 125 levels may affect the diagnosis [11]. Pongsuvareeyakul et al. found a sensitivity of the frozen section for borderline mucinous tumors to be 67.2 % [12].

Diagnosis of germ cell tumors and sex cord stromal tumors is sometimes difficult on the frozen section as they can mimic surface epithelial tumors. In our study, there were 20 cases of germ cell tumors, all which were mature teratomas and 8 cases of sex cord stromal tumors (4 cases each of granulosa cell tumor and fibro-thecoma). Adult granulosa cell tumors are considered as low-grade malignancy, and initial surgical management depends on the stage of the disease. All of 4 cases in our study were limited to the ovary at initial diagnosis, and there was no evidence of recurrence on follow-up.

The main limitation of the frozen section lies in the accurate diagnosis of borderline ovarian tumors especially of the mucinous category. In a larger retrospective review of 622 ovarian tumors, 52 cases were rendered borderline on frozen section. Terms like at least borderline were also used. Out of these 52 cases, concordance with final diagnosis was seen in 37 cases with an accuracy of 71.15 %. Under-diagnosis occurred in 12 cases and over-diagnosis in 3 cases [13]. Under-diagnosis in case of borderline ovarian tumors usually occurs as a result of under-sampling. As WHO criteria for the diagnosis of borderline mucinous tumors is quantitative (>10 % of tumors examined showing atypical proliferative features in the absence of frank invasion), it can be misinterpreted in limited sections available on the frozen section. The incidence of over-diagnosis is rare with some examples showing tangential cutting leading to false impression of invasion. In our study, there were two cases in which under-diagnosis was rendered. One of them was of mucinous borderline tumor which was labeled as benign on the frozen section. On further sampling of the tumor after formalin fixation, it turned out to be borderline with >10 % tumor showing epithelial proliferation with nuclear atypia and mitotic activity meeting the criteria of borderline malignancy. The other case was diagnosed as borderline serous tumor on the frozen section due to atypical epithelial proliferation. On final histology, focal areas of invasion were noted occupying >3 mm area and therefore the diagnosis was changed to serous carcinoma. This highlights the importance of sampling for ovarian tumors especially for those of benign and borderline category. Both our cases with disagreements on final paraffin sections seem to be due to sampling errors. Therefore, we suggest that matriculate sampling is needed in ovarian tumors specifically from areas where wall appears thick or there is evidence of solid growth.

## Conclusions

In conclusion, we found a high sensitivity and specificity of the frozen section for the diagnosis of ovarian tumors and to determine its malignant potential. Therefore, it should always be used when the preoperative diagnosis is not definite to govern extent of surgical resection. However, under-diagnosis can occur in tumors of borderline category, especially those of mucinous histology, which can be minimized by increased sampling on the frozen section.

### Consent

Written informed consent was obtained from the patients for publication of the data. A copy of the written consent is available for review by the Editor-in-Chief of this journal. The ethics committee of Liaquat National hospital approved the study.
